# Recent Progresses in the Treatment of Osteoporosis

**DOI:** 10.3389/fphar.2021.717065

**Published:** 2021-07-22

**Authors:** Shan-Shan Li, Shi-Hao He, Peng-Yu Xie, Wei Li, Xin-Xin Zhang, Tian-Fang Li, Dai-Feng Li

**Affiliations:** ^1^ Department of Rheumatology, The First Affiliated Hospital of Zhengzhou University, Zhengzhou, China; ^2^ Department of Orthopaedics, The First Affiliated Hospital of Zhengzhou University, Zhengzhou, China; ^3^ Department of Magnetic Resonance Imaging, Henan Key Laboratory of Functional Magnetic Resonance Imaging and Molecular Imaging, The First Affiliated Hospital of Zhengzhou University, Zhengzhou, China

**Keywords:** osteoporosis, antiresorptive drugs, anabolic drugs, wnt signaling pathway, bone formation

## Abstract

Osteoporosis (OP) is a chronic bone disease characterized by aberrant microstructure and macrostructure of bone, leading to reduced bone mass and increased risk of fragile fractures. Anti-resorptive drugs, especially, bisphosphonates, are currently the treatment of choice in most developing countries. However, they do have limitations and adverse effects, which, to some extent, helped the development of anabolic drugs such as teriparatide and romosozumab. In patients with high or very high risk for fracture, sequential or combined therapies may be considered with the initial drugs being anabolic agents. Great endeavors have been made to find next generation drugs with maximal efficacy and minimal toxicity, and improved understanding of the role of different signaling pathways and their crosstalk in the pathogenesis of OP may help achieve this goal. Our review focused on recent progress with regards to the drug development by modification of Wnt pathway, while other pathways/molecules were also discussed briefly. In addition, new observations made in recent years in bone biology were summarized and discussed for the treatment of OP.

## Introduction

The pathogenesis of osteoporosis (OP) may result from different factors such as aging, glucocorticoid use and heavy alcohol consumption. Aging is often associated with reduced bone mass, abnormal microstructure and fragile fracture, which poses a tremendous challenge to the medical communities (Compston et al., 2019; Tatangelo et al., 2019). Healthy bone has dynamic and balanced formation and resorption. Thus, two types of drugs, namely, anti-resorptive and pro-formative, are used to treat OP. Anti-resorptive drugs take their effect by interfering normal functions of osteoclasts. This type of drugs includes bisphosphonates (BPPs), estrogen, selective estrogen receptor modulators (SERMs), the antibodies against receptor activator of nuclear factor κB (NF-κB) ligand (RANKL), etc. While BPs can increase bone mineral density (BMD), they may decrease the flexibility of bone, increasing fracture risk (Russell et al., 2007). As such, pro-formative (anabolic) drugs have attracted wide attention in recent years (Langdahl, 2020). However, the concerns remain with regard to their cost-effectiveness, the efficacy in cortical bone, the potential adverse effects on endocrine and cardiovascular systems (Martin, 2016; Miller et al., 2016; Fuggle et al., 2020). Mounting data indicates a critical role of Wnt signaling pathway in bone formation, and novel therapeutics may be discovered through modifying inhibitors or activators of this pathway (Lerner and Ohlsson, 2015). Our review summarized the working mechanisms of both types of drugs and discussed the potential outcomes of some investigative drugs with the focus on Wnt pathway.

## Methods

We searched PubMed for combinations of the following indexed subject headings (MeSH): Osteoporosis, antiresorptive drugs, anabolic drugs, Wnt signaling pathway, bone formation.

### Skeletal Biology

**FIGURE 1:**
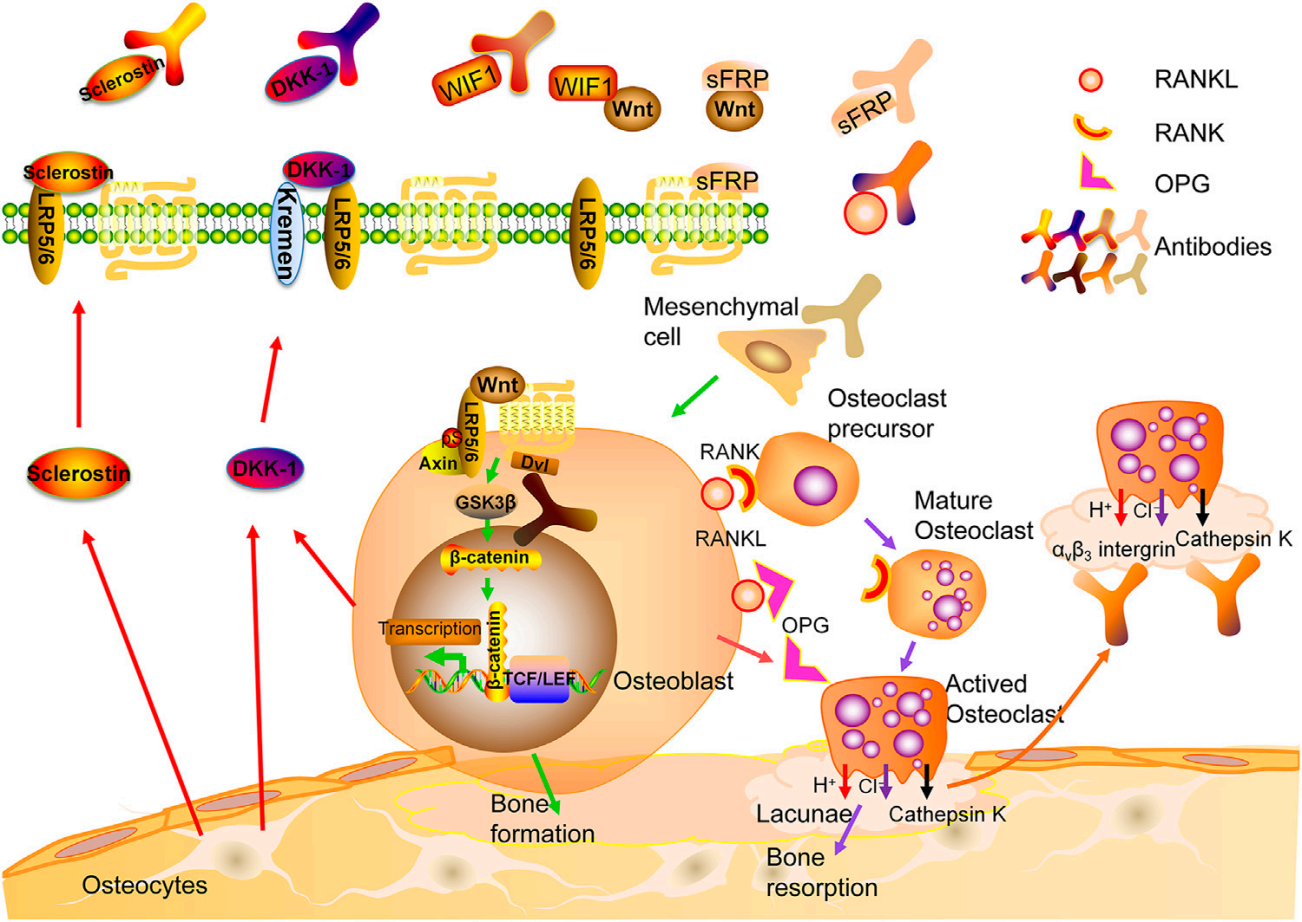
Bone remodeling and therapeutic targets for osteoporosis. RANK: Receptor activator of nuclear factor-kb; RANKL: RANK ligand; OPG: osteoprotegerin.

Osteoclasts (OCs) are derived from hematopoietic stem cells and formed by the fusion of monocytes through complicated mechanisms. Multiple factors are involved in the differentiation, activation and survival of OCs including receptor activator of NF-κB ligand (RANKL), a molecule produced by different types of cells including osteoblasts (OBs), OCs, bone marrow stromal cells, lymphocytes, etc. In an acidic microenvironment formed by the sealing zone of OCs, cathepsin K is the most important enzyme to degrade non-mineral components of bone such as collagen type I (Col-I). The attachment of OCs on bone surface is mediated by integrins, mainly α_v_β_3_ (Lewiecki, 2011). OBs are derived from mesenchymal stem cells (MSCs). Mature OBs produce osteoid consisting of Col-I and non-collagenous proteins. Mineralization of osteoid ensues and osteoblasts are embedded in bone, referred to as osteocytes (OCTs) (Lewiecki, 2011; Eastell et al., 2016). While OCTs were thought to be quiescent cells, several lines of evidence suggest they are active participants of bone metabolism. They can perceive mechanical loading signal and be regulated by hormones to coordinate coupling processes of formation and resorption mediated by OCs and OBs. In addition, OCTs are the major source of sclerostin, a potent inhibitor of Wnt pathway (Eastell et al., 2016) (Figure 1).

### Anti-Resorptive Durgs

While some anti-resorptive agents such as BPPs, estrogen and denosumab, have been proven effective in some patients (Cheng et al., 2020), investigative agents targeting the molecules of resorption lacuna hold great promises.

#### Currently Available Anti-resorptive Drugs

While BPPs are commonly used agents for primary and secondary osteoporosis to increase BMD, they do affect the flexibility of bone (Russell et al., 2007). They may cause atypical subtrochanteric fractures and are not recommended for young patients (Van den Wyngaert et al., 2006; Russell et al., 2007). Another concern is osteonecrosis of jaw, particularly, for those who will have dental procedures in the near future (Van den Wyngaert et al., 2006; Russell et al., 2007). Estrogen replacement therapy may increase cardiovascular events, venous thromboembolism and breast cancer (Rossouw et al., 2002; Almeida et al., 2017). While selective estrogen receptor modulators (SERMs) have a reduced risk of breast cancer (Cummings et al., 1999; Lindsay et al., 2009; Cummings et al., 2010), their efficacy is lower than estrogen (Ettinger et al., 1999; Silverman et al., 2008; Reid, 2015).

Denosumab is a fully human IgG2 monoclonal antibody (mAb) against the ligand of the RANK receptors on the surface of osteoclast precursors (RANKL) (Lacey et al., 2012). Binding of RANKL to RANK activates multiple signaling pathways. The binding of TNF receptor-associated factors (TRAFs) to specific sites in the cytoplasmic domain of RANK is crucial for differentiation and survival of OCs (Boyce and Xing, 2008). Osteoprotegerin (OPG), a decoy receptor, may compete with RANKL for the binding to RANK (Kearns et al., 2008; Infante et al., 2019). Bone mass was significantly reduced in OPG-knockout mice, while it is increased after overexpressing OPG (Nakamura et al., 2003) (Figure 1).

Previous studies have demonstrated that denosumab can improve the structure and thickness of cortical bone, and reduce the porosity of trabecular bone although it decelerates the turn-over of bone (Genant et al., 2013; Zebaze et al., 2016). Clinical trials have shown that in the first year, it may reduce the risk of vertebral and non-vertebral fractures (Cummings et al., 2009). While prolonged treatment leads to continuous increase of BMD, the risk of infection also increases. Besides, atypical femoral fractures and osteonecrosis may occur although the incidence is low (Bone et al., 2017). More studies are warranted to maximize its efficacy and minimize its adverse events. Of note, after withdrawal of denosumab, the BMD rapidly declines with subsequent increase in fracture risk. Thus, additional anti-resorptive drugs are required to maintain the treatment outcomes (Rizzoli et al., 2010; Collison, 2017).

#### Anti-resorptive Drugs Under development

##### Targeting the Molecules of Resorption Lacuna

Cathepsin K, the primary cysteine protease secreted by mature OCs, is involved in the degradation of Col-I and other bone matrix proteins (Costa et al., 2011). The observations made from different animal models have shown that inhibiting cathepsin K decreases osteoclastic bone resorption and increases bone formation (Gowen et al., 1999; Duong et al., 2016a; Duong et al., 2016b). The selective cathepsin K inhibitors, such as Odanacatib (Langdahl et al., 2012; Statham and Aspray, 2019), ONO-5334 (Engelke et al., 2014) and MIV-711 (Lindström et al., 2018; Conaghan et al., 2020), have been shown to reduce bone resorption and continuously increases BMD at multiple sites. Unfortunately, due to the adverse events, especially stroke, further development is restricted (Mullard, 2016; McClung et al., 2019). One explanation is that cathepsin K deficiency may disrupt the blood-brain barrier *via* AKT-mTOR-VEGF signaling, causing neurological deficits and neuron apoptosis (Zhao et al., 2019). Other concern is the rapid loss of functions after cessation of treatment (Eastell et al., 2014). Further, chloride channel-7 (ClC-7) and cathepsin K coexists and works synergistically in the ruffled border of OCs. The damage of ClC-7 results in severe OP, possibly due to the defect in bone degradation caused by the inability to acidify the sealing zone (Kornak et al., 2001). However, a CIC-7 inhibitor, N53736, showed a long-term anti-resorptive effect in ovariectomized (OVX) rats (Schaller et al., 2004), thus, more studies are needed.

As integrin α_v_β_3_ mediates the attachment of OCs onto bone matrix proteins, it is reasonable to hypothesize that inhibiting the subunit of this integrin may prevent bone resorption. In different animal models of induced osteoporosis, α_v_β_3_ integrin antagonists such as L-000845704 and HSA-ARLDDL significantly increase the BMD (Murphy et al., 2005; Lin et al., 2017). In addition, a dual-specific protein, macrophage colony-stimulating factor (M-CSF_RGD_), may bind to and inhibit both c-FMS and α_v_β_3_ integrin. *In vitro* and *in vivo* studies shows that it inhibits OCs activity (Zur et al., 2018). These results indicate that targeting molecules adjacent to resorption lacuna may pave a new way to the treatment of OP.

All anti-resorptive agents mentioned above are listed in Table1.

**TABLE 1:** Currently available and promising anti-resorptive agents.

Classification	Category	Medicine	Property	Adverse events/limitations
Antiresorptive drugs	Bisphosphonates (Khosla et al., 2012; McClung et al., 2013)	Alendronate	i) An analog of inorganic pyrophosphate with a high affinity for bone hydroxyapatite ii) Able to prevent endogenous bone mineralization and inhibit functions and survival of osteoclasts. (Fleisch, 1998)	i) Osteonecrosis of the jaw ii) Atypical subtrochanteric femoral fractures (Van den Wyngaert et al., 2006; Russell et al., 2007)
		Risedronate		
		Ibandronate		
		Zoledronic acid		
	Estrogen (Rossouw et al., 2002; Eastell et al., 2016)	Estrogen	i) Directly enhancing osteogenic differentiation of MSCs and suppressing osteoblasts apoptosis ii) Up-regulating the expression of RANKL in osteoblasts and the production of OPG, IGF1 and TGF-β, thus, interfering downstream signal in osteoclasts. (Hofbauer and Schoppet, 2004; Almeida et al., 2017) iii) Indirect alteration the expression of estrogen- responsive target genes, giving rise to bone turnover. (Weitzmann and Pacifici, 2006; Eastell et al., 2016) iv) Potential bone formation due to the connection among estrogen, mechanical loading, sclerostin and osteocytes. (Lee et al., 2003; Modder et al., 2011)	i) Lingering risk on cardiovascular ii) venous thromboembolic events iii) breast cancer (Rossouw et al., 2002; Almeida et al., 2017)
	Selective estrogen receptor modulators	Raloxifene (Ettinger et al., 1999)	Interaction with ERs and a range of tissue-specific agonist and antagonist effects	Compared to estrogen, without adverse effects on the breast. (Cummings et al., 1999; Cummings et al., 2010)
		Bazedoxifene (Silverman et al., 2008)		
	RANKL inhibitor	Denosumab (Cummings et al., 2009)	Blocking RANKL-RANK interaction by neutralizing RANKL to inhibit bone resorption. (Lacey et al., 2012)	i) Osteonecrosis of the jaw ii) Atypical subtrochanteric femoral fractures (Bone et al., 2017)
Promising anti-resorptive drugs	Cathepsin K inhibitor	Odanacatib	i) Prevention of bone resorption without affecting bone formation and continuous increase of spinal BMD in postmenopausal women. (Statham and Aspray, 2019) ii) Robust anti-fracture effect with good tolerability (Rizzoli et al., 2016)	Stroke (McClung et al., 2019)
		ONO-5334	Robust and persistent increase of trabecular and integral BMD. (Engelke et al., 2014)	The effect on biochemical markers was rapidly reversible on treatment cessation (Eastell et al., 2014)
		MIV-711	Significant reduction of the biomarkers of bone resorption and cartilage loss. (Lindström et al., 2018)	The RCT trails were conducted only in osteoarthritis currently (Conaghan et al., 2020)
	α_v_β_3_ integrin antagonist	L-000845704	Significant increase in spinal BMD. (Murphy et al., 2005)	Only several preclinical studies *in vitro* and animal study
		HSA-ARLDDL	Prevention of ovariectomized-induced reduction in cancellous bone volume, bone surface, and trabecular number in rats (Lin et al., 2017)	
		M-CSF_RGD_	i) A dual-specific protein able to bind to and inhibit both c-FMS and α_v_β_3_ integrin ii) Suppressing osteoclast activity (Zur et al., 2018)	
	Chloride channel-7 inhibitor	N53736	i) Overcoming the defect in bone degradation due to the inability to acidify the sealing zone ii) A long-term anti-resorptive effect in ovariectomized rats (Schaller et al., 2004)	No clinical trials

### Anabolic Drugs

#### Non-wnt Related Anabolic Drugs

Teriparatide (a recombinant human PTH 1–34) may enhance bone formation by promoting osteoblast differentiation and functions. In the early stage of treatment, intermittent administration of teriparatide stimulates bone formation on cancellous, endosteal, and periosteal surfaces. Its effects on cortical bone vary at different sites (Martin, 2016). Randomised controlled trials (RCTs) show a higher efficacy of teriparatide than risedronate regarding the incidence of vertebral and non-vertebral fractures (Neer et al., 2001; Kendler et al., 2018). Similarly, Abaloparatide, a synthetic analogue of PTHrP, reduces the fracture risk in these sites. In addition, Abaloparatide has a higher efficacy in the increment of BMD and lower incidence of hypercalcaemia than Teriparatide (Leder et al., 2015a; Miller et al., 2016). Further, it is superior to Teriparatide and Alendronate with regard to the reduction of fracture risks (Miller et al., 2016; Reginster et al., 2019; Leder et al., 2020). Compared with Teriparatide, Abaloparatide has higher affinity to PTH1R and is able to specifically stimulate osteogenesis. Nevertheless, there is a controversy about whether these effects are due to decreased bone resorption or increased bone formation (Reginster et al., 2018). Although no increased risk of osteosarcoma is observed in patients, laboratory studies have shown a dose-dependent increase of osteosarcoma in rats treated with either Teriparatide or Abaloparatide (Vahle et al., 2004; Jolette et al., 2017). Therefore, it is recommended that the duration of Teriparatide treatment should be limited to 24 months (Andrews et al., 2012).

All currently available anabolic agents are in Table 2. In detailed discussion of romosozumab and blosozumab will be presented in the following section.

**TABLE 2:** Available anabolic drugs.

Category	Medicine	Property	Adverse events
Parathyroid hormone receptor agonist	Teriparatide (Neer et al., 2001) Abaloparatide (Miller et al., 2016)	i) Acting on PTH1R on the surface of osteoblasts and resulting in the induction and transient signalling of intracellular cAMP. (Yang et al., 2007) ii) Mitogenic property for osteoblast and driving bone formation by generating ATP from both glycolysis and mitochondrial respiration. (Karner and Long, 2018) iii) Indirectly enhancing Wnt signaling through a variety of other signaling pathways, including IGF1, FGF2 and BMPs. (Estell and Rosen, 2021) iv) Binding toLRP6 to form a complex, leading to increased β-catenin levels and the expression of osteogenic genes. (Wan et al., 2008) v) Suppressing sclerostin and promoting osteoblast-driven bone formation. (Bellido et al., 2013) vi) Inducing transactivation of Runx2 and osteoblast differentiation *via* the cAMP and/or protein kinase A (PKA) pathway. (Swarthout et al., 2002)	i) Hypercalcaemia ii) Osteosarcoma (Vahle et al., 2004; Miller et al., 2016; Jolette et al., 2017)
mAb against sclerostin	Romosozumab	i) It is pro-anabolic but anti-resorptive by neutralizing sclerostin	i) Cardiovascular events
			ii) Osteoarthritis (Bouaziz et al., 2015)
	Blosozumab		Phase 3 results are awaited

#### Wnt Signaling Pathways and Potential Agents and Targets

##### Wnt Signaling Pathway Activation

**FIGURE 2:**
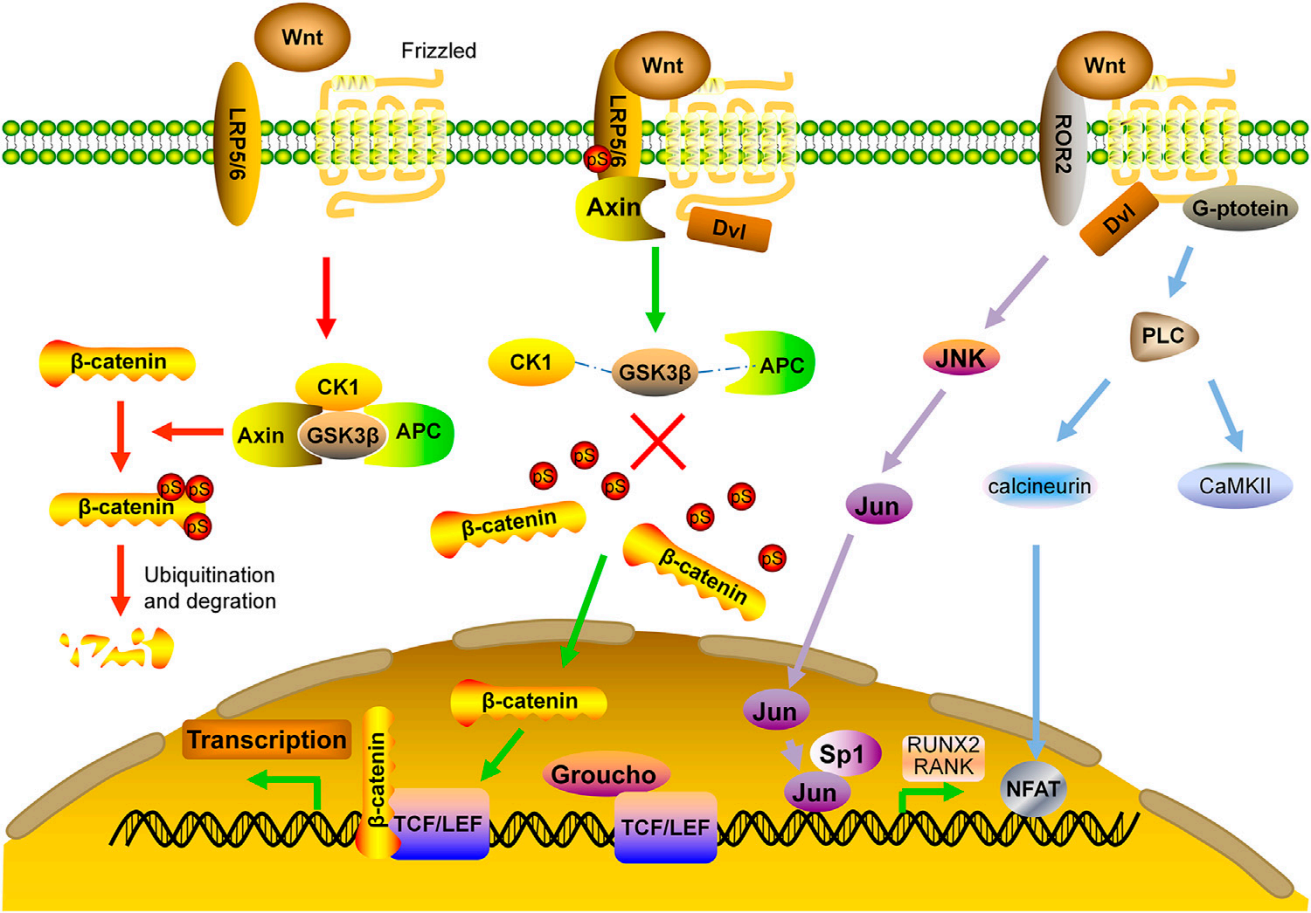
Wnt signaling pathway in bone formation. APC: adenomatous polyposis *coli*; CaMKII: calcium calmodulin-mediated kinase II; CK1: casein kinase one; Dvl: dishevelled; FZD: frizzled; GSK3β: glycogen synthase kinase 3β; JNK: c-Jun N-terminal kinase; LRP: low density lipoprotein receptor related protein; NFAT: nuclear factor of activated T cells; RUNX2: runt-related transcription factor 2; TCF/LEF: T-cell specific transcription factor/lymphoid enhancer binding factor.

The Wnts are secreted, lipid-modified glycoproteins. After binding to their cell surface receptors, they can take effect *via* either canonical or non-canonical pathways. The canonical pathway is predominant in bone formation. The receptors of different Wnts in the canonical pathway consist of the low-density lipoprotein receptor related protein (LRP) single-pass transmembrane co-receptors 5/6 and the seven-transmembrane signaling receptor Frizzled (FZD) (Ng et al., 2019). In the downstream of this pathway, there is a destruction complex containing Axin, adenomatous polyposis *coli* (APC), casein kinase 1 (CK1) and glycogen synthase kinase 3β (GSK3β). In the absence of Wnt ligands, β-catenin is phosphorylated by GSK3β with subsequent ubiquitination and degradation (MacDonald et al., 2009; Clevers and Nusse, 2012). Upon Wnt binding, dishevelled (Dvl) disassembles the destruction complex, preventing phosphorylation of β-catenin. Non-phosphorylated β-catenin accumulates in the cytoplasm, and translocates to the nucleus whereby it forms a nuclear complex with T-cell specific transcription factor/lymphoid enhancing factor (TCF/LEF) transcription factor, which then causes the recruitment of co-activators and induction of gene transcription (Tolwinski and Wieschaus, 2004; MacDonald et al., 2009; Baron and Kneissel, 2013) (Figure 2).

Non-canonical Wnt signaling pathway is independent of β-catenin, instead, it takes effects by activating the heterotrimeric G-proteins and protein kinase C (PKC), which inhibits MSC differentiation toward adipocyte lineage and stimulates the nuclear factor of activated T cells (NFAT) to regulate bone formation and bone resorption (Kohn and Moon, 2005). Non-canonical Wnt signaling also induces Rho-or c-Jun N-terminal kinase (JNK)-dependent changes in the actin cytoskeleton, which facilitates Jun and Sp1 transcription factor to regulate the bone related molecules such as RANK and Runx2 (Veeman et al., 2003; Krishnan et al., 2006; Amjadi-Moheb and Akhavan-Niaki, 2019) (Figure 2).

##### Wnt Signaling and Bone Anabolism

Wnt signaling pathway enhances bone anabolism by inducing osteoblast differentiation, suppressing osteoclastogenesis and preventing adipogenesis. Expression of Wnt target genes such as Runx2, induces differentiation of MSC precursors to osteoblastic lineage, promoting bone formation (Gaur et al., 2005; Davis and Zur Nieden, 2008). Activation of Wnt pathway increases glycolysis in OBs, providing then the energy needed for collagen synthesis and matrix mineralization (Karner and Long, 2017). Remodeling on cortical bone is increased markedly due to activation of OBs on both the cortical and trabecular surface. In addition, canonical Wnt signaling inhibits bone resorption by increasing OPG production (Boyce et al., 2005). A study showed that bone formation was reduced in mice deficient with either FZD receptor or β-catenin although the production of OPG was not changed. It is postulated that Wnt signaling may repress osteoclastogenesis in a mechanism different from RANK/RANKL/OPG axis (Albers et al., 2013). Moreover, through enhancing phosphorylation of β-catenin, sclerostin facilitates adipogenesis (Fairfield et al., 2017). In a mouse model of myeloma, mAb against sclerostin increased bone mass and decreased the number of bone marrow adipocytes (McDonald et al., 2017).

##### Epigenetic Mechanisms Regulating Wnt Signaling

Epigenetic modification of some important molecules in Wnt pathway may affect bone metabolism. Bone biopsy from postmenopausal women with osteoporotic fractures shows a higher serum level of sclerostin. Increased CpGs methylation in the proximal region of the promoter of the *Sost* gene reduces the inhibitory effect of slcerostin on Wnt pathway, thereby enhancing bone formation (Reppe et al., 2015). Previous studies have shown that histone acetylation of Wnt gene promoter is reduced owing to the inhibition of lysine acetyltransferase 2A (GCN5) expression, resulting in suppression of Wnt signaling (Jing et al., 2018). In addition, a histone-lysine N-methyltransferase enzyme, an enhancer of zeste homolog 2 (EZH2), suppresses osteogenic differentiation of MSCs. Inhibition of EZH2 prevents bone loss (Dudakovic et al., 2015; Dudakovic et al., 2016). Overexpression of histone deacetylases 5 (HDAC5) downregulates the expression of sclerostin in osteocytes (Wein et al., 2015; Wein et al., 2016). miRNAs also play an important role in regulation of Wnt signaling (Amjadi-Moheb and Akhavan-Niaki, 2019). MiR-27a decreases OC differentiation and bone resorption through a binding site in the 3′-untranslational region of APC (Guo et al., 2018). During osteogenic differentiation of human stromal/stem cells, by inhibiting secreted frizzled-related proteins (sFRPs), dickkopf (DKK) and sclerostin, the signal amplification circuit between miR-218 and Wnt/β-catenin signals is established to drive Wnt-related transcription and OB differentiation (Hassan et al., 2012; Zhang et al., 2014). Other miRNAs, such as miR-29, miR-542-3p and miR-335-5p, can also regulate different molecules in Wnt pathway (Kapinas et al., 2010). Furthermore, miR-16-2*, by regulating the expression of Runx2, may be involved in OB differentiation, matrix mineralization and pathogenesis of OP (Duan et al., 2018).

##### Wnt Antagonists

Inhibition of canonical Wnt signaling pathway can be done by neutralizing Wnt ligands or blocking their binding to the receptor LRP/FZD. Wnt antagonists such as Wnt inhibitory factor 1 (WIF-1) and sFRPs prevent ligands binding to their cognate receptor. WIF-1 is structurally similar to the extracellular portion of the Derailed/Ryk class of transmembrane Wnt receptors. It may inhibit Wnt activity during OB differentiation and maturation (Vaes et al., 2005; Canalis, 2013). However, overexpression of WIF-1 activates canonical Wnt signaling and results in the loss of self-renewal potential of resident hematopoietic stem cells, suggesting it is not an optimal target for regulation of bone formation (Schaniel et al., 2011).

sFRPs block Wnt signaling by interacting with Wnts or FZD. Previous studies have demonstrated that sFRP1 is a negative regulator of cancellous bone formation and overexpression of sFRP4 in OBs reduces bone mass (Kawano and Kypta, 2003; Bodine et al., 2004; Nakanishi et al., 2008). Somewhat surprisingly, deletion of sFRP4 decreases the thickness of cortical bone, possibly by activating non-canonical signaling (Kiper et al., 2016; Chen et al., 2019), suggesting that fine-tuning the concentrations of sFRPs is needed before future trials.

Sclerostin and DKK1 block Wnt/β-catenin pathway by binding to LRP5/6. Sclerostin is mainly expressed by OCTs, and its binding to LRP5/6 inhibits bone formation and enhanced bone resorption (Li et al., 2005). Besides, osteocyte-produced sclerostin is transported to bone surface or adjacent OCTs, where it inhibits osteoblast-mediated bone formation, and increases bone resorption by OCs as well as osteocytic osteolysis by stimulating RANKL production and downregulating OPG expression (Ke et al., 2012; Appelman-Dijkstra and Papapoulos, 2018). Sclerostin may also play a role in other signaling pathways. An *in vivo* study has shown that mechanical stress activates Wnt pathway by down-regulating sclerostin expression, whereas upregulation of sclerostin expression in unloaded bone leads to bone loss (Robling et al., 2008). Of note, one underlying mechanism for anabolic effects of intermittent administration of PTH on bone is to inhibit sclerostin expression (Bellido et al., 2013).

DKK1 is a secreted glycoprotein produced by OCTs and OBs, and it contains the cysteine-rich domains that can bind to LRP5/6. DKK1 coupled with transmembrane receptor Kremen may form a complex with LRP to inhibit Wnt signaling (Mao et al., 2002; Pinzone et al., 2009). Further, DKK1 antagonizes osteoblastogenesis from MSCs and Wnt-mediated OB differentiation. Increased production of RANKL and decreased production of OPG mediated by DKK1 causes net bone loss (Pinzone et al., 2009).

##### Drugs Related to the Wnt Signaling Pathway

Romosozumab, a humanized antibody that neutralizes sclerostin, has been approved by the FDA for OP treatment. Several trials have demonstrated that it significantly increases BMD and decreases new vertebral and non-vertebral fractures (McClung et al., 2014). However, romosozumab did not improve the fracture-healing-related outcomes of hip fractures (Schemitsch et al., 2020). A recent study showed that romosozumab induced a transient bone formation in the first 2 months and a sustained suppression of bone resorption for up to 12 months (Chavassieux et al., 2019). As the anabolic effects of anti-sclerostin therapy are short-lived, it is reasonable to hypothesize that intermittent and short-term treatment with romosozumab might be just as effective as the continuous treatment for 12 months (Cosman et al., 2016; Saag et al., 2017). Sustainable BMD gains can be achieved by sequential therapy with romosozumab followed by denosumab (McClung et al., 2018; Kendler et al., 2019; Lewiecki et al., 2019). The STRUCTURE trial has shown that romosozumab is superior to Teriparatide with regard to increase in bone mass and strength (Langdahl et al., 2017). Romosozumab is not recommended for patients with a previous myocardial infarction or other cardiovascular events because of potential adverse effects (Lewiecki et al., 2018). Two meta-analyses showed inconsistent results in terms of the increase in cardiovascular risk (Bovijn et al., 2020; Lv et al., 2020). One explanation is that sclerostin is expressed in aortic vascular smooth muscle and can inhibit angiotensin II-induced atherosclerosis. Systemic blockade of sclerostin may affect the remodeling process in the cardiovascular system (Krishna et al., 2017; Asadipooya and Weinstock, 2019). A study showed that the second course of treatment with romosozumab had similar effects as the treatment in the first year (McClung et al., 2020), however, the BMD increments were smaller than those observed during the first year (McClung et al., 2018; Kendler et al., 2019). The duration of romosozumab treatment remains a matter of debates. At the moment, it is well accepted that the treatment should be no longer than 12 months (Table 3).

**TABLE 3:** Clinical trials assessing the efficacy of romosozumab in osteoporosis.

Trial and year	Rationale/Question Behind study	Design	Conclusion
Frame, 2016 (Cosman et al., 2016)	Compare the incidence of fractures between romosozumab-to-denosumab group and the placebo-to-denosumab group in postmenopausal women with osteoporosis	Subcutaneous injections of romosozumab (210 mg monthly) or placebo for 12 months, followed by subcutaneous injection of denosumab (60 mg every 6 months) for 12 months	The rates of fractures were significantly lower in the romosozumab group than in the placebo group
Arch, 2017 (Saag et al., 2017)	Compare the incidence of fractures between romosozumab-to-alendronate group and the alendronate-to-alendronate group in postmenopausal women with osteoporosis	Randomly assigned patients to receive monthly subcutaneous romosozumab (210 mg) or weekly oral alendronate (70 mg) for 12 months, followed by open label alendronate	Compared to alendronate alone, romosozumab treatment for 12 months followed by alendronate resulted in a significantly lower risk of fracture
STRUCTURE, 2017 (Langdahl et al., 2017)	Evaluated the effects of romosozumab or teriparatide on BMD in women with postmenopausal osteoporosis transitioning from bisphosphonates therapy	Patients were randomly assigned to receive subcutaneous romosozumab (210 mg once monthly) or subcutaneous teriparatide (20 µg once daily) after at least 3 years of oral bisphosphonates	Compared to teriparatide, bone mass and strength increased to a greater extent in women treated with romosozumab
Bridge, 2018 (Lewiecki et al., 2018)	Evaluate the safety and efficacy of romosozumab in men with osteoporosis	The subjects were randomized to receive romosozumab 210 mg subcutaneously monthly or placebo for 12 months	Compared with placebo,treatment with romosozumab for 12 months increased BMD significantly and was well tolerated

Blosozumab, another mAb against sclerostin, has shown to be well-tolerated in completed phase 1 and phase 2 trials. It increased BMD in a dose-dependent manner. Phase 3 results are awaited with excitement (McColm et al., 2014; Recker et al., 2015). To the best of our knowledge, no clinical trials are conducted to compare the efficacy in BMD increment between blosozumab and romosozumab.

AbD09097, a new anti-sclerostin agent, was examined *in vitro* about its effect on bone formation (Boschert et al., 2016). Combination of mechanical loading and anti-sclerostin antibodies in mice caused higher bone formation than either anti-sclerostin antibodies or mechanical loading alone (Morse et al., 2018). This study suggests that a combination of pharmacotherapy and physiotherapy may achieve sustained improvement of bone quality and persistent reduction of fracture risk. The effectiveness of the available nanocarriers, mesoporous silica nanoparticles (MSNs) loading with osteostatin and SOST siRNA is evaluated, and its subcutaneous injection up-regulated the expression of osteogenic related genes, thus, improving bone microarchitecture. More studies are needed before clinical application of such delivery system (Mora-Raimundo et al., 2021).

Preclinical studies have been performed to test the effect of mAb to DKK1. It improved BMD improvement in OVX rodents, whereas only a minimal improvement was observed in OVX monkeys (Glantschnig et al., 2011; Li et al., 2011). Notably, a bispecific antibody directed at both sclerostin and DKK1 has been generated and shown a more significant BMD improvement than mono-antibody in OVX rats (Florio et al., 2016). Because of the concern of off-target effects of DKK1 inhibitors in non-skeletal tissues, no clinical trials are currently going on.

Lithium, a GSK3β inhibitor, can activate Wnt-β-catenin pathway. Mice treated with lithium chloride (LiCl) lowered fracture risk. It stimulated bone formation, but did not affect bone resorption (Clement-Lacroix et al., 2005; Vestergaard et al., 2005). A newly-developed GSK3β inhibitor rapidly increased the number of OBs and decreased the number of OCs, resulting in a significant increase in bone volume, trabecular number and trabecular thickness (Clement-Lacroix et al., 2005; Amirhosseini et al., 2018). LY294002, an inhibitor of phosphatidylinositol-3-kinase-protein kinase B (PI3K-AKT) signaling pathway, can inhibit OC differentiation. However, both LiCl and LY294002 are highly toxic at conventional doses (Huang et al., 2018). Low doses of combined LiCl and LY294002 not only promote bone formation and inhibit bone resorption, but also are more effective in the treatment of OP than either single compound (Bai et al., 2019). Additionally, ample phytochemicals, such as Baicalin, Aspp049, Wedelolactone, Ursolic acid, may enhance GSK3β phosphorylation, Runx2 expression, and nuclear translocation of β-catenin, thus, enhancing osteogenic differentiation and bone formation (Manandhar et al., 2020). Despite these results, lacking bone specificity and potential off-target effects hinder further development of GSK3β inhibitors for the treatment of OP (Hall et al., 2015).

Animal study was conducted to evaluate the effect of sFRP1 inhibitors on OP and these included imino-oxothiazolidines, diarylsulfone sulfonamides and N-substituted piperidinyl diphenylsulfonyl sulfonamides (WAY-316606). The results showed increased OB activation and bone formation (Claudel et al., 2019) Further, miR-542-3p and miR-1-3p inhibited sFRP1 expression and induced OB differentiation (Zhang et al., 2018; Gu et al., 2020). Based on these findings, miRNA-based therapies targeting sFRPs are likely to become novel approach to prevent and treat osteoporosis.

The possible therapeutic targets mentioned above have been identified in Figure 1.

#### Interaction of Wnt Pathway With Other Signaling Pathways

Bone morphogenetic proteins (BMPs) belong to the TGF-β superfamily. Among them, BMP-2 up-regulates the expression of Runx2 through Smad pathway, leading to enhanced bone formation. In addition, BMP-2 inhibits the activity of E3 ubiquitin ligase to prevent degradation of β-catenin and up-regulates the expression of WNT3A, WNT1, and LRP, which causes accumulation of β-catenin and activation of Wnt signaling pathway, thereby, increasing bone formation (Wu et al., 2016).

PI3K-AKT pathway can be activated in OBs by various growth factors. This pathway positively regulates Wnt signaling by stabilizing β-catenin and deactivating GSK3β. Previous studies have demonstrated that AKT may form a complex with BMP-2, and its related downstream signals are essential regulators for OB differentiation and endochondral ossification. AKT knockout mice had shorter bones and delayed bone ossification (Ulici et al., 2009). In addition, AKT phosphorylation by upstream kinase mTORC2 may cause accumulation of β-catenin both in cytoplasm and nucleus (Sarbassov et al., 2005; Rybchyn et al., 2011). One study shows that miR-483-5p mimic activates PI3K-AKT signaling pathway and affects cell viability, with significant down-regulation of the expressions of OPG, Runx2 and BMP2. Consistently, LY294002 and miR-483-5p inhibitor reverse these effects and increase BMD and biomechanical parameters for anabolism (Zhao et al., 2021). Moreover, interaction of MAPK pathway with Wnt signaling not only regulates survival and apoptosis of OCs, but also enhances BMP-2 expression and bone formation (Tang et al., 2008; Chen et al., 2014). A study demonstrates that miR-182-5p inhibits the expression of adenylyl cyclase isoform 6 (ADCY6) and activation of the Rap1/MAPK signaling pathway. Down-regulation of miR-182 promotes OB proliferation and differentiation (Pan et al., 2018).

Other pathways may also have cross-talks with Wnt pathway. For example, Adenosine Monophosphate Activated Protein kinase (AMPK) may activate canonical Wnt signaling pathway and up-regulate the expression of BMP-2 (Zhao et al., 2010). AMPK also phosphorylates HDAC5, resulting in the activation of Wnt signaling (Zhao et al., 2011).

**FIGURE 3:**
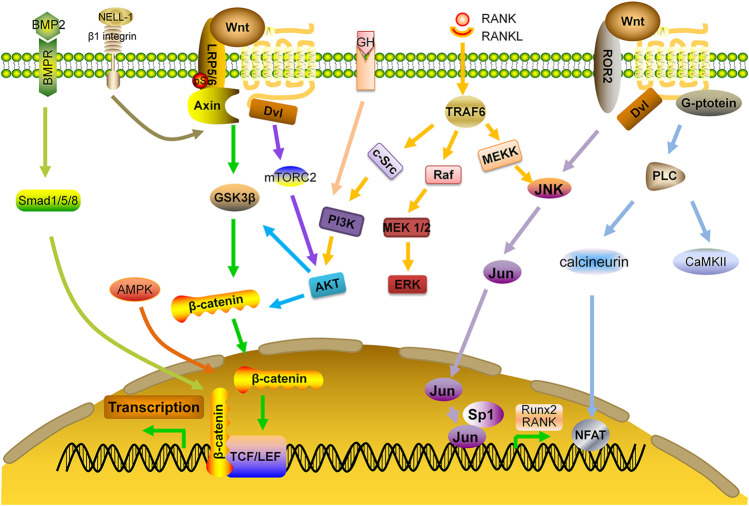
Interaction of Wnt signaling with other signaling pathways. AKT: protein kinase B; AMPK: adenosine monophosphate-activated protein kinase; BMP: bone morphogenetic protein; BMPR: BMP receptor; ERK: extracellular signal-regulated kinase; GF: growth factor; MEK: mitogen-activated protein kinase; NELL-1: NEL-like protein one; PI3K: phosphoinositide 3-kinase; Smad: small mothers against decapentaplegic; TRAF: TNF receptor-associated factors.

Protein kinase C-binding protein NELL-1 is an osteoinductive growth factor that can bind to β1-integrin on the surface of bone cells. It not only activates canonical Wnt pathway and regulates the activity of Runx2, but also has a reciprocal impact on BMP-2 signaling by enhancing osteogenesis and inhibiting adipogenesis (Zhang et al., 2011; Shen et al., 2016; Pakvasa et al., 2017). In OVX mice, NELL-1 down-regulated RANKL expression and up-regulated OPG expression, leading to enhanced bone formation and decreased number of OCs (James et al., 2015). Delivering NELL-1 to vertebrae of osteoporotic sheep or femurs of OVX rats can improve the regeneration of cortical and trabecular bone (James et al., 2016; James et al., 2017). Additional studies are needed to determine the feasibility and efficacy of this protein as an anabolic agent (Figure 3).

### Combined and Sequential Therapies

#### Combined and Sequential Therapies

The effect of the most anti-osteoporotic drugs, except for BPs, is not sustainable on bone metabolism. In some cases, an overshooting response may occur when they are discontinued. In particular, withdrawal of anabolic drugs often causes rapid bone loss and increases risk of fractures. Further, anabolic treatment with Teriparatide or Abaloparatide may incite secondary stimulation of bone resorption. It is reasonable to postulate that the effects of bone-forming treatments may be improved and maintained with combined or sequential treatments. Ongoing clinical studies on combination and sequential therapies are summarized in Tables 4, 5. It is now unanimously accepted that the administration of bone-forming agents should be followed by an anti-resorptive agent. In addition, the evaluation of the effectiveness of combined therapies is still ongoing.

**TABLE 4:** Combination therapies.

Anabolic agents	Anti-resorptive drugs	Methods	Conclusions
PTH (1–84)	Alendronate (Black et al., 2003)	Randomly assigned patients to daily treatment with parathyroid hormone (1–84) (100 µg), alendronate (10 mg), or both for 12 months	i) There was no evidence of synergy between parathyroid hormone and alendronate ii) The anabolic effects of parathyroid hormone may be reduced when use of alendronate simultaneously
PTH (1–84)	Ibandronate (Schafer et al., 2012)	Participants received either 6 months of concurrent PTH and ibandronate, followed by 18 months of ibandronate (concurrent) or two sequential courses of 3 months of PTH followed by 9 months of ibandronate (sequential) over 2 years	i) BMD did not increase more than with either treatment alone ii) Concurrent monthly ibandronate may blunt the effects of PTH(1–84)
Teriparatide	Zoledronic Acid (Cosman et al., 2011)	Randomly assigned patients to receive a single intravenous infusion of zoledronic acid 5 mg plus daily teriparatide 20 mg *via* subcutaneous injection, zoledronic acid alone, or placebo infusion plus daily teriparatide 20 mg for 1 year	A beneficial effect of co-administration of teriparatide and zoledronic acid treatment was shown as compared to teriparatide or zoledronic acid monotherapy
Teriparatide	Denosumab (Tsai et al., 2013; Tsai et al., 2019)	Patients were assigned in a 1:1:1 ratio to receive 20 µg teriparatide daily, 60 mg denosumab every 6 months, or both	Combined teriparatide and denosumab increased BMD more than either agent alone
		Participants were randomly assigned (1:1) to receive teriparatide 20 µg (standard dose) or 40 µg (high dose) daily for 9 months. At 3 months, both groups were started on denosumab 60 mg every 6 months for 12 months	Combined treatment with teriparatide 40 µg and denosumab increased BMD more than standard combination therapy

**TABLE 5:** Sequential therapies.

Initial agents	Subsequent agents	Methods	Conclusions
Teriparatide	Denosumab	Subjects were switched from both the combination and teriparatide groups to denosumab, and subjects in the denosumab group were switched to teriparatide. In all groups, 24 months of additional treatment were given. (Leder et al., 2015b)	In postmenopausal osteoporotic women switching from teriparatide to denosumab, BMD continued to increase
Denosumab	Teriparatide		In postmenopausal osteoporotic women switching from denosumab to teriparatide results in progressive or transient bone loss
Abaloparatide	Alendronate (Bone et al., 2018)	Patients who had been randomized to either placebo or abaloparatide (80 µg daily) for 18 months were subsequently treated with oral alendronate (70 mg weekly) for an additional 24 months	Sequential abaloparatide followed by alendronate had a greater reducion in the risk of fractures and BMD increased more
Romosozumab	Denosumab (Lewiecki et al., 2019)	Patients received romosozumab or placebo (month 0–12) followed by denosumab (month 12–36)	BMD were further augmented and fracture risk was reduced by switching from romosozumab to denosumab

## Discussion

Pathogenesis of OP, especially, in postmenopausal women, is multifaceted. Improved understanding of skeletal biology will help us identify new therapeutic targets with maximal efficacy and minimal adverse effects. Our review summarized recent progress in molecular mechanisms and major signaling pathways involved in bone homeostasis and OP pathogenesis. The approaches to prevent OP include anti-resorption by suppressing OC activity and pro-formation by enhancing OB functions. OCTs, used to be thought as the quiescent cells embedded in bone matrix, have been demonstrated to be critical in the regulation of OCs and OBs activities, warranting in-depth understanding of OCT biology. Taking cost-effectiveness into account, the mainstay of current treatments is still anti-resorptive drugs, particularly, BPPs, in most developing countries. However, as they can incorporate into bone and prevent bone resorption, normal dynamic remodeling process, especially in young adults, is interrupted, which may reduce the flexibility of bone (Russell et al., 2007).

We focused on Wnt pathway because accumulating data indicated a pivotal role of this pathway in bone metabolism. Comparing with TGF-β and NF-kB pathways, Wnt signaling pathway is more complicated and more targets are available for modifications both extra- or intracellularly. Elegant studies from different animal models have laid a solid foundation for new drugs development by regulating Wnt pathway. In the canonical Wnt pathway, the modification of the destruction complex is under intensive studies. For example, manipulating the activity GSK3β may enhance anabolic property of OBs (Amirhosseini et al., 2018). Similarly, regulating the expression of Axin-2 and APC may cause constitutive activation of canonical pathway to promote bone formation (Nusse and Clevers, 2017; Huang et al., 2019). However, the specificity and their potential off-target risks of some newly developed agents for modifying Wnt pathways have been halted after phase 1 or phase 2 trials. Delivery systems using peptides or chemicals with high affinity to bone are expected to overcome these drawbacks (Guan et al., 2012; Zur et al., 2018; Rammal et al., 2019). Bi-specific Wnt mimetic targeting both FZD and LRP has demonstrated a rapid and robust effect on bone building and correction of bone mass deficiency (Fowler et al., 2021), however, more studies are needed before preclinical and clinical trials of this agent. Besides, a cell/gene therapy in combination with miRNA manipulation may become effective treatment for osteoporosis. For example, hybrid vector engineered OVX-BMSCs were used to lower miR-140*/miR-214 levels, promote osteogenesis and enhance bone quality (Li et al., 2016). Further, the utilization of nanocarriers-based therapies that interact Wnt pathway hold great promise as novel therapy for osteoporosis. In contrast, because of the complexity and multiple alternatives of non-canonical Wnt pathway, there is a scarcity of data regarding the role of non-canonical Wnt pathway in bone metabolism. New targets may be identified after extensive studies of non-canonical Wnt pathway (Lerner and Ohlsson, 2015).

Other research interests include the mechanisms and treatment of the loss of cortical bone as it is more closely related to osteoporotic fractures. Aging is also an important factor for OP. Targeting the senescent cells by modification of the aging-related genes or pharmacological methods, such as Janus kinase (JAK) inhibitor, have both anti-resorptive and pro-formative effects on bone (Farr et al., 2017). In addition, more investigations should be carried out to elucidate the mechanism for bone erosion in some autoimmune diseases, especially, in rheumatoid arthritis (Minisola et al., 2021). Of note, osteoporosis is common in patients with ankylosing spondyloarthritis (AS), even in young males (Sambrook and Geusens, 2012). A recent study showed that miR-96 may promote osteoblast differentiation and bone formation in AS mice *via* Wnt signaling activation by binding to sclerostin (Ma et al., 2019). Further, the major pathway mediating glucocorticoid induced bone loss need to be further dissected in order to preserve their anti-inflammatory activity, but avoid the harmful skeletal effect of this most commonly used drug in autoimmune rheumatic diseases (Hartmann et al., 2016).

## Conclusion

Although significant progresses have been made in recent years, the prevention and treatment of osteoporosis and the related fractures remain an unmet medical need. In-depth understanding of molecular events in the pathogenesis of osteoporosis including epigenetic regulation of Wnt pathway may facilitate the development of new drugs with better efficacy and less side effects.

## Glossary

AMPK: Adenosine Monophosphate Activated Protein kinase
APC: adenomatous polyposis coli
AS: ankylosing spondyloarthritis
BMD: bone mineral density
BMP: Bone morphogenetic protein
BMSCs: bone marrow stem cells
BPPs: bisphosphonates
CIC-7: Chloride channel-7
CK1: casein kinase 1
Col-I: collagen type I
DKK1: Dickkopf 1
EZH2: enhancer of zeste homolog 2
FZD: Frizzled;
GCN5: lysine acetyltransferase 2A
GSK3β: phosphorylating enzyme glycogen synthase kinase 3β
HDAC5: histone deacetylases 5
JAK: Janus kinase
JNK: c-Jun N-terminal kinase
LiCl: lithium chloride
LRP: low density lipoprotein receptor related protein
M-CSF: macrophage colony-stimulating factor
MSCs: mesenchymal stem cells
NELL-1: NEL-Like molecule-1;
NFAT: nuclear factor of activated T cells
OBs: osteoblasts
OCs: osteoclasts
OP: osteoporosis
OPG: osteoprotegerin
OCTs: osteocytes
OVX: ovariectomized
PI3K-AKT: phosphatidylinositol-3-kinase-protein kinase B
PKC: protein kinase C
RANKL: receptor activator of nuclear factor κB (NF-κB) ligand
Runx2: runt-related transcription factor 2
SERMs: selective estrogen receptor modulators
sFRPs: secreted frizzled-related proteins
TCF/LEF: T-cell specific transcription factor/lymphoid enhancing factor
TRAFs: TNF receptor-associated factors
WIF-1: Wnt inhibitory factor 1
